# Current and emerging strategies for myopia control: a narrative review of optical, pharmacological, behavioural, and adjunctive therapies

**DOI:** 10.1038/s41433-025-03949-1

**Published:** 2025-08-01

**Authors:** Furqan A. Maulvi, Ditixa T. Desai, Parthasarathi Kalaiselvan, Dinesh O. Shah, Mark D. P. Willcox

**Affiliations:** 1 School of Optometry and Vision Science, University of New South Wales, Sydney, NSW Australia; 2 Maliba Pharmacy College, Uka Tarsadia University, Surat, India; 3 Department of Chemical Engineering and Department of Anesthesiology, University of Florida, Gainesville, FL USA

**Keywords:** Vision disorders, Refractive errors

## Abstract

Myopia has become a leading cause of visual impairment globally, with a rapidly increasing prevalence among children, particularly in urbanised areas of East and Southeast Asia. High and pathologic myopia can lead to irreversible complications, including retinal detachment, glaucoma, and myopic maculopathy. This narrative review synthesises current and emerging strategies for myopia control as of 2025, integrating evidence from optical, pharmacological, behavioural, and surgical domains. Multifocal contact lenses, orthokeratology, and defocus-incorporated spectacles are effective in modulating axial elongation. Low-dose atropine remains a cornerstone pharmacologic therapy with consistent efficacy. Behavioural interventions, such as increased outdoor time, provide preventive benefits and are endorsed in school-based programs. Repeated low-level red-light (RLRL) therapy represents a novel, non-invasive option with growing support. Surgical approaches, while corrective rather than preventive, are relevant in advanced cases. The review also compares intervention efficacy, discusses the rationale for combination therapies, and highlights the need for individualised, age-appropriate strategies. Key challenges include treatment variability, limited long-term data, and barriers to adherence and access. Future directions involve personalised risk prediction, global implementation frameworks, and public health engagement. A multimodal, patient-centred approach is essential to reduce the lifelong burden of myopia.

## Introduction

Myopia, or nearsightedness, is a refractive condition characterised by the elongation of the eyeball, causing distant objects to appear blurred as light rays focus anterior to the retina [1]. While traditionally considered a benign and easily correctable condition, myopia has now emerged as a major global public health concern due to its rapidly increasing prevalence and its association with sight-threatening complications in high and pathologic forms [2]. These include myopic maculopathy, retinal detachment, glaucoma, and choroidal neovascularization, which significantly contribute to irreversible vision loss, particularly in East and Southeast Asia [3]. This article is structured as a narrative review, aiming to synthesise current clinical evidence on myopia control strategies based on key clinical trials, meta-analyses, and reviews published from 2000 to 2025.

The World Health Organization (WHO) and the International Myopia Institute (IMI) have recognised myopia as a growing epidemic, projecting that nearly 50% of the global population will be myopic and ~10% will have high myopia by 2050 [4, 5]. Environmental and behavioural factors—such as increased screen time, excessive near work, and reduced outdoor activity—have been identified as major contributors to this trend, particularly in children and adolescents [6]. While genetic predisposition plays a role, the steep increase over a short period highlights the influence of modifiable risk factors.

In recent years, the clinical approach to myopia has shifted from a passive model of optical correction to an active strategy targeting the prevention of onset and slowing of axial elongation [7]. A range of interventions has been developed and investigated, including specialised spectacle and contact lenses, low-dose atropine eye drops, orthokeratology, and behavioural modifications such as increasing time spent outdoors [8]. More recently, repeated low-level red-light (RLRL) therapy has emerged as a promising non-invasive intervention, supported by encouraging results from randomised clinical trials [9]. Surgical interventions, although not commonly used for myopia control, play a role in the management of severe or pathologic myopia.

Given the breadth of available interventions and rapidly evolving evidence, this review aims to provide a comprehensive, narrative synthesis of current and emerging strategies for myopia management in 2025. Emphasis is placed on mechanisms of action, clinical efficacy, safety, age-specific considerations, and practical implementation. In addition, we highlight challenges in adherence, accessibility, and long-term outcome evaluation, particularly in paediatric populations. The goal is to assist clinicians, researchers, and public health policymakers in understanding the multifaceted landscape of myopia control and to encourage the adoption of individualised, multimodal approaches based on current best practices. This review is structured thematically, covering epidemiology, pathophysiology, classification, and the spectrum of interventions—including optical, pharmacological, behavioural, and surgical options. Emerging therapies such as RLRL and combination protocols are discussed in detail. Finally, we explore gaps in the current knowledge base and propose directions for future research and clinical translation.

## Epidemiology and global burden of myopia

The prevalence of myopia has risen dramatically in recent decades, making it one of the most common ocular disorders worldwide. According to Lee et al. (2024), earlier projections estimating that by 2050 nearly 50% of the global population will be myopic and 10% will have high myopia may significantly overstate future prevalence due to limitations in data representativeness and modelling assumptions. They emphasise the need for more nuanced, region-specific forecasting models to better reflect real-world trends and guide public health interventions [10].

The epidemiological distribution of myopia is highly skewed by geography, urbanisation, and lifestyle. The most alarming rates have been observed in East and Southeast Asia, where up to 80–90% of high school graduates are myopic and 10–20% progress to high myopia [11]. Countries such as China, South Korea, Japan, Singapore, and Taiwan have experienced a rapid rise, largely attributed to increased educational pressures, prolonged near work, and limited time spent outdoors [12]. For example, longitudinal data from China indicate that the prevalence of severe myopia increased from 7.9 to 16.6% over 15 years in urban youth populations [13, 14]. Japan similarly reports a high burden, with myopia-related macular degeneration contributing significantly to visual impairment [15].

Although the epidemic is most pronounced in Asia, similar upward trends have been reported in Western nations [4]. Studies from the United States, Australia, and parts of Europe indicate a doubling of myopia prevalence in recent decades, albeit from a lower baseline [16**–**18]. Ethnic disparities remain evident; Asian populations demonstrate a higher propensity for both onset and progression compared to Caucasian or African populations. For instance, pathologic myopia affects an estimated 1–3% of Asians versus approximately 1% of Caucasians [19].

The burden of myopia extends beyond refractive correction to include increased risk of ocular comorbidities [20]. High and pathologic myopia are strongly associated with irreversible structural changes in the posterior segment of the eye, including scleral thinning, chorioretinal atrophy, and tractional maculopathy. These complications significantly increase the risk of legal blindness and lead to long-term dependency and decreased quality of life. In older adults, myopic maculopathy is now one of the leading causes of blindness in some developed regions of Asia [11]. Furthermore, the economic burden is substantial, encompassing direct costs of corrective lenses and clinical monitoring, as well as indirect costs associated with vision loss, reduced productivity, and caregiving [21].

Public health responses to this growing epidemic have been inconsistent. While some regions, notably Taiwan and China, have implemented school-based interventions promoting outdoor activity, such initiatives are lacking in many parts of the world [22]. The need for early detection, widespread education, and accessible therapeutic interventions is critical to halting the progression of myopia at a population level. In summary, myopia is no longer a benign refractive condition but a public health priority with growing global and economic consequences. Coordinated efforts in surveillance, clinical management, and preventive strategies are urgently needed to address its expanding burden.

## Pathophysiology and classification of myopia

Myopia, or nearsightedness, arises when the eye’s axial length exceeds the focal length required to project images directly onto the retina, resulting in light focusing anterior to the retinal plane. Although traditionally considered a simple refractive error, myopia is now recognised as a multifactorial condition involving complex physiological, genetic, and environmental interactions that drive pathological ocular elongation [23]. In recent years, insights from both clinical and experimental studies have refined our understanding of its underlying mechanisms, which are crucial for developing effective interventions.

The predominant cause of myopic progression is excessive axial elongation. Visual signals, particularly peripheral hyperopic defocus—where light focuses behind the peripheral retina—trigger compensatory mechanisms that stimulate ocular growth. This phenomenon is mediated by neuromodulators such as dopamine, nitric oxide, and retinoic acid, which influence biochemical pathways in the retina and sclera [24]. These signals alter scleral extracellular matrix remodelling, leading to thinning and elongation of the posterior globe. Experimental evidence from animal models supports the theory that manipulating peripheral retinal image quality can directly influence axial length growth [25].

Advances in imaging modalities, such as optical coherence tomography (OCT) and magnetic resonance imaging (MRI), have revealed key structural changes associated with progressive and pathological myopia. These include scleral thinning, choroidal atrophy, and the development of posterior staphyloma [26]. High and pathologic myopia are also associated with tractional complications, such as myopic macular degeneration and foveoschisis, which contribute to permanent vision loss in advanced stages.

Classification of myopia can be approached from several clinical angles. Based on refractive severity, myopia is typically categorised as low (<–3.00 dioptres), moderate (–3.00 to –6.00 dioptres), and high (>–6.00 dioptres) [27]. Pathologic myopia, however, refers to cases where degenerative structural changes in the posterior segment are present, regardless of refractive error magnitude. Anatomically, myopia is divided into axial myopia—driven by globe elongation—and refractive myopia, which results from increased curvature or refractive index of the cornea or lens [28]. Axial myopia is the most prevalent and carries a greater risk of progression and associated pathology.

Age of onset further stratifies myopia into congenital, childhood or youth-onset, early adult-onset, and late-onset categories. Congenital myopia is often linked with systemic conditions or genetic syndromes. Youth-onset myopia typically manifests during school-age years and is strongly associated with environmental risk factors such as excessive near work and limited outdoor activity. Late-onset myopia is more commonly linked to occupational visual demands and may also result from nuclear sclerosis in aging lenses [29, 30].

In terms of progression patterns, myopia may be stationary, temporarily progressive, or permanently progressive. Temporarily progressive cases often stabilise in the early twenties, whereas permanently progressive myopia continues to worsen over time and is frequently linked with axial elongation and structural ocular damage. Identification of these patterns is critical for determining prognosis and guiding treatment decisions. While hereditary factors contribute to baseline risk, environmental influences are increasingly recognised as dominant drivers of the global myopia epidemic. The growing body of evidence supports the concept that early-life exposure to high near-visual demand and inadequate outdoor light exposure accelerates onset and progression. Understanding these pathophysiological mechanisms and classification systems provides the foundation for designing personalised and timely interventions to reduce the burden of myopia [31, 32].

## Current Strategies for myopia management

### Optical interventions

Optical interventions represent one of the most established and accessible strategies in the clinical management of myopia, particularly in children and adolescents [8]. These interventions not only correct refractive errors but are also specifically designed to influence ocular growth by modifying peripheral defocus. The concept underlying most optical strategies is based on the evidence that peripheral hyperopic defocus stimulates axial elongation, whereas peripheral myopic defocus inhibits it. Modern lens technologies have sought to harness this principle to slow myopia progression effectively [33].

Traditional single-vision lenses (SVLs), though effective in correcting central refractive errors, do not alter the peripheral retinal image profile and have little to no effect on the progression of myopia. Indeed, studies have shown that children wearing SVLs continue to experience axial elongation despite optimal correction [34, 35]. This has led to the development of various advanced lens designs, including multifocal soft contact lenses, orthokeratology lenses, and specialised spectacle lenses.

Multifocal soft contact lenses (MFCLs) have gained significant traction as a non-invasive method to induce peripheral myopic defocus [36]. These lenses, particularly those with high add power, have demonstrated substantial reductions in the rate of axial elongation. The BLINK study, a landmark randomised controlled trial, showed that children using +2.50 dioptre add-power MFCLs experienced a 43% slower progression over three years compared to those using SVLs [34]. Similar results have been observed with extended depth-of-focus (EDOF) lenses, which offer smoother power gradients to maintain both visual quality and therapeutic defocus [37].

Spectacle-based options such as Defocus Incorporated Multiple Segments (DIMS) lenses and Highly Aspherical Lenslet Target (HALT) technology have further expanded the armamentarium. DIMS lenses incorporate a central zone for clear vision surrounded by annular segments that provide constant myopic defocus. A two-year randomised trial in Hong Kong demonstrated a 52% reduction in myopia progression and a 62% reduction in axial elongation with DIMS lenses [38]. Similarly, Stellest™ lenses with HALT technology have shown significant reductions in axial elongation through their multiple aspherical lenslets arranged in concentric patterns [39].

Orthokeratology (Ortho-K) involves overnight wear of specially designed rigid gas-permeable lenses that temporarily reshape the corneal surface to correct refractive error and induce a peripheral myopic defocus during daytime [40]. Numerous studies, including the ROMIO trial, have confirmed that Ortho-K can slow axial elongation by 30–60% over one to two years [41]. This method is particularly attractive for active children and those with moderate myopia but requires rigorous compliance and regular monitoring to minimise the risk of complications, such as microbial keratitis.

The efficacy of these optical interventions depends on several factors, including age, baseline refraction, lens design, and adherence. While contact lens-based therapies tend to offer superior myopia control compared to spectacles, spectacle lenses remain more suitable for younger children due to their ease of use and safety profile. Increasingly, clinicians are also exploring combined approaches—such as MFCLs or Ortho-K used alongside low-dose atropine—to enhance efficacy [42]. In summary, optical interventions have become the cornerstone of modern myopia management. Their ability to provide both vision correction and therapeutic control makes them particularly valuable in paediatric populations, where early and sustained intervention is critical.

### Pharmacological therapies

Pharmacological treatment, particularly with low-dose atropine, has emerged as one of the most effective interventions for slowing myopia progression in children. Unlike optical strategies that primarily act through modulation of visual stimuli, pharmacological agents influence biochemical signalling pathways that govern ocular growth, particularly axial elongation. Among available options, atropine—a non-selective muscarinic receptor antagonist—has demonstrated the most consistent clinical efficacy [43, 44].

Early studies using 1% atropine showed a marked reduction in myopia progression but were limited by significant side effects, including photophobia, blurred near vision, and poor tolerability [45]. These limitations prompted the evaluation of lower concentrations. The landmark Atropine for the Treatment of Myopia (ATOM) 2 study compared 0.01%, 0.1%, and 0.5% atropine in children and found that 0.01% provided a favourable balance between efficacy and minimal side effects. This low-dose formulation has since become widely accepted in clinical practice, offering a 50–60% reduction in progression with a minimal rebound effect upon discontinuation [46, 47].

More recently, the Low-Concentration Atropine for Myopia Progression (LAMP) study conducted in Hong Kong further refined the dosage debate [48, 49]. It reported that 0.05% atropine was the most effective among the concentrations tested (0.01%, 0.025%, 0.05%), achieving the greatest reduction in axial elongation over two years, with acceptable tolerability. Nevertheless, 0.01% remains the most commonly prescribed due to its safety profile and broad global use [50].

The mechanism by which atropine slows myopic progression is not entirely dependent on its effect on accommodation, as previously assumed. Instead, current evidence suggests involvement of non-accommodative pathways, potentially acting through retinal or scleral muscarinic receptors, dopamine release modulation, and nitric oxide signalling. These mechanisms inhibit the remodelling of the scleral extracellular matrix and suppress axial elongation [51].

Population-specific responses to atropine have also been observed. While East Asian populations have shown consistent benefits, some studies from Western countries, including a recent U.S. trial, reported limited efficacy of 0.01% atropine in European children, suggesting that genetic, environmental, or dosing differences may influence outcomes [52]. This has prompted the exploration of tailored approaches based on individual risk profiles, ethnicity, and progression rates.

Another pharmacological agent, pirenzepine, a selective M1 receptor antagonist, showed early promise in reducing myopic progression by approximately 40% in randomised trials. However, it failed to gain regulatory approval due to formulation challenges and limited commercial interest, and its development has since been discontinued. Pharmacological therapy is typically initiated in children demonstrating progressive myopia—defined as a change of ≥0.5 dioptres per year—especially when optical interventions are insufficient or not tolerated. Regular monitoring of refraction and axial length is essential during treatment, and therapy is often continued for 2–3 years. Importantly, tapering the dose gradually upon discontinuation reduces the risk of rebound progression [53, 54]. In conclusion, low-dose atropine represents a safe, well-tolerated, and effective pharmacological tool for myopia control, especially when tailored to individual needs. As our understanding of its mechanisms deepens, and with the potential for combination strategies, pharmacologic therapy will remain a cornerstone in the multifaceted management of paediatric myopia.

### Behavioural and environmental interventions

Behavioural and environmental factors play a critical role in the development and progression of myopia, particularly in children. Increasing evidence from epidemiological and interventional studies supports the hypothesis that lifestyle-related visual demands—especially prolonged near work and reduced exposure to outdoor light—significantly contribute to the onset and acceleration of myopia [55]. As such, non-pharmacological, behaviour-based strategies have gained attention as cost-effective and universally applicable tools for myopia prevention, especially at the community and school level.

Among these, increasing the duration and frequency of outdoor activity has shown the most robust protective effect against myopia onset [56]. Exposure to natural light is believed to trigger the release of retinal dopamine, a neuromodulator that inhibits ocular elongation. Bright outdoor light also reduces accommodative strain by encouraging long-distance gaze, thereby minimising the stimulus for axial growth. The protective benefit appears to be dose-dependent, with at least 2 h per day of outdoor time recommended for young children to meaningfully reduce risk [57].

The most compelling clinical evidence comes from randomised controlled trials in East Asia. In a large school-based trial in Guangzhou, China, children assigned an additional 40 min of outdoor time per school day showed significantly lower incidence of new-onset myopia after one year compared to controls [58]. Similarly, a cluster-randomised trial in Taiwan demonstrated that children exposed to ambient light intensities exceeding 1000 lux during recess exhibited slower myopic shifts and axial elongation [59, 60]. These results have prompted the implementation of national myopia prevention programs in several Asian countries, integrating outdoor activity into daily school routines.

Despite the strong preventive effect on onset, the role of outdoor activity in slowing progression once myopia has already developed remains less clear. Some studies suggest modest benefits, while others report minimal or no effect on axial length in already myopic children. Nevertheless, its low cost, non-invasive nature, and overall benefits for physical and mental health make outdoor exposure a highly recommended lifestyle adjustment.

In contrast, excessive near work, such as prolonged reading or screen time, has been associated with a higher risk of myopia [61]. While the relationship is complex and potentially confounded by other factors, it is generally advised to limit continuous near tasks to no more than 30–40 min at a time, followed by visual breaks or distance viewing. The “20-20-20 rule”—looking at something 20 feet away for 20 s every 20 min—has been suggested as a simple method to reduce accommodative stress and visual fatigue [62].

Other environmental modifications, such as optimising indoor lighting, maintaining proper reading distance (at least 30–40 cm), and encouraging posture awareness, may also contribute to reducing visual strain, though the evidence supporting these measures is less robust. A comparative summary of the mechanisms, age suitability, efficacy, and potential side effects of commonly used interventions is presented in Table 1. In conclusion, behavioural and environmental interventions, particularly increased outdoor time, represent practical and scalable approaches to mitigate the rising burden of myopia. While not a substitute for clinical therapies in progressive cases, these strategies serve as essential adjuncts and preventive tools, especially when implemented early in life. Public health campaigns, parental awareness, and school-based initiatives are vital to promote these behavioural modifications on a population level.

**Table 1:** Summary of current interventions for myopia control.

Intervention	Mechanism of Action	Target Age Group	Efficacy (% Reduction in Progression)	Notable Side Effects
Low-dose Atropine (0.01–0.05%)	Muscarinic receptor blockade reducing axial elongation	Children (5–16 years)	30–70%	Photophobia, near blur (rare at low doses)
Orthokeratology (Ortho-K)	Corneal reshaping inducing peripheral myopic defocus	Children/Adolescents (7–16 years)	30–60%	Risk of infection (keratitis), lens discomfort
Multifocal Soft Contact Lenses (MFCLs)	Peripheral myopic defocus via multi-zone optics	Children/Adolescents (7–16 years)	30–50%	Discomfort, handling difficulties
DIMS Spectacle Lenses	Peripheral myopic defocus with lenslets	Children (6–14 years)	40–60%	Minimal; visual adaptation phase
HALT/EDOF Spectacle Lenses	Peripheral myopic defocus with aspheric designs	Children (6–14 years)	35–50%	Minimal; possible adaptation period
Repeated Low-Level Red-Light (RLRL) Therapy	Photobiomodulation affecting retinal/scleral signalling	Children (7–13 years)	30–60%	Compliance dependent; safety being monitored
Increased Outdoor Activity	Dopamine release from bright light exposure; reduced near work	Preschool and school-aged children	20–25% (onset only)	None; adherence needed
Posterior Scleral Reinforcement (PSR)	Mechanical support to prevent posterior globe expansion	Adolescents/Adults with pathologic myopia	May reduce axial elongation (~20–40%)	Surgical risks: haemorrhage, fibrosis
Refractive Surgery (LASIK/SMILE/ICL)	Corneal/lens alteration to correct refractive error	Adults with stable myopia	No effect on progression, only correction	Surgical risks: dry eye, glare, halos
Intervention	Mechanism of Action	Target Age Group	Efficacy (% Reduction in Progression)	Notable Side Effects

## Emerging and adjunct therapies

### Repeated Low-Level Red-Light (RLRL) Therapy

RLRL therapy has recently emerged as a promising non-pharmacological and non-optical strategy for controlling myopia progression, particularly in children. This therapy involves the use of low-intensity red light—typically at a wavelength of ~650–660 nm—administered to the eye in brief, repeated sessions using specialised desktop or wearable light-emitting devices. Unlike traditional interventions, RLRL is hypothesised to modulate ocular growth through mechanisms that are still under investigation but appear to be distinct from those involved in defocus regulation or muscarinic signalling [62, 63].

The clinical interest in RLRL therapy was initially sparked by observations that light exposure at specific wavelengths and intensities could influence axial elongation in animal models [64]. These findings were translated into human trials, most notably in China, where RLRL therapy was evaluated in randomised controlled settings. A pivotal multicentre study involving over 260 children aged 8–13 years reported significantly slower axial elongation and myopia progression in those receiving twice-daily RLRL therapy compared to controls using single-vision spectacles. Notably, the therapy was well tolerated, with no reported structural or functional damage to ocular tissues [65, 66]. Another double-blind, sham-controlled trial further confirmed the efficacy of RLRL. In this study, children undergoing full-power RLRL exposure (2 × 3 min per day, 5 days a week) demonstrated significantly less axial elongation and refractive shift over six months compared to a matched control group receiving inactive light treatment. These effects were maintained in follow-up assessments up to 12 months. Importantly, no adverse events or retinal changes were observed on optical coherence tomography (OCT) or fundus imaging, supporting the safety of the intervention [64].

The proposed mechanism of action for RLRL involves the stimulation of mitochondrial activity and increased ATP production in retinal cells, potentially influencing scleral remodelling and choroidal blood flow. It has also been speculated that RLRL may modulate circadian pathways or dopamine release, both of which have established roles in eye growth regulation. However, these hypotheses remain to be fully validated in mechanistic studies. Despite its early promise, several questions remain regarding the optimal dosing regimen, long-term durability of effect, and generalisability across populations. Most published trials have been conducted in East Asian children, where baseline myopia progression rates are high. Data from Western or multi-ethnic populations are currently limited. Moreover, adherence and practical implementation, especially in school or home settings, require further consideration. Device accessibility, cost, and regulatory approval will also influence the scalability of RLRL as a mainstream clinical tool.

In summary, RLRL therapy represents a novel and potentially transformative approach to myopia control. Its non-invasive nature and promising efficacy make it an attractive adjunct to existing interventions. While longer-term studies and broader clinical validation are still needed, early results suggest that RLRL could play a valuable role in future multimodal strategies for managing childhood myopia.

### Combination therapy

Combination therapy for myopia control—most commonly involving pharmacological and optical modalities—is gaining increased attention as a strategy to maximise treatment efficacy by targeting multiple pathways of ocular growth regulation [64]. This approach is especially relevant for children exhibiting rapid progression or inadequate response to monotherapy. The most studied and widely adopted combination to date is the concurrent use of low-dose atropine with orthokeratology (Ortho-K), although other pairings, such as atropine with multifocal soft contact lenses or specialised spectacle lenses, are also under active investigation [42].

The rationale behind combination therapy stems from the distinct mechanisms of action associated with different treatment types. Atropine, a muscarinic receptor antagonist, is believed to inhibit axial elongation through biochemical modulation of scleral remodelling and retinal signalling pathways. In contrast, Ortho-K acts by inducing a temporary anterior flattening of the cornea and peripheral myopic defocus, thereby modifying visual input to suppress eye growth. By addressing both neuromodulatory and optical feedback mechanisms, combination therapy theoretically provides additive or synergistic effects [42].

Several studies have demonstrated that combining low-concentration atropine (usually 0.01% or 0.025%) with Ortho-K results in superior control of axial elongation compared to Ortho-K alone [67]. A two-year randomised controlled trial in East Asia found that children receiving both treatments had a 28% greater reduction in axial elongation compared to those using Ortho-K alone [68]. Importantly, the addition of low-dose atropine did not appear to significantly impact corneal reshaping or increase the risk of adverse effects when properly monitored. A recent meta-analysis further supported these findings, indicating that the dual regimen consistently outperformed monotherapies in terms of both refractive and axial length outcomes.

Combination therapy with atropine and multifocal soft contact lenses or defocus-incorporated spectacle lenses is also showing promise. Preliminary reports suggest that these combinations may enhance efficacy without introducing significant new safety concerns. For example, the concurrent use of 0.01% atropine and DIMS (Defocus Incorporated Multiple Segments) lenses has shown additive effects in reducing axial elongation in children with high-risk progression profiles. However, large-scale trials are still needed to validate these outcomes and identify optimal combinations [69, 70].

Despite the potential advantages, combination therapy also presents challenges. Increased treatment complexity can affect adherence, especially in younger children. Cost may also be a limiting factor, particularly when both pharmaceutical and optical devices are needed. Furthermore, there is currently no consensus on standardised protocols for initiating, monitoring, and tapering combination regimens. Clinicians must therefore exercise individualised judgement, considering baseline progression rates, age, parental preferences, and prior response to monotherapy.

Safety remains a key consideration in combination therapy. While low-dose atropine is generally well tolerated, the use of higher concentrations in combination regimens has been associated with increased risk of side effects, such as photophobia and blurred near vision. Therefore, higher atropine doses (>0.05%) are not typically recommended in dual approaches. In conclusion, combination therapy offers a promising avenue for enhancing myopia control, particularly in children with aggressive progression or suboptimal response to single interventions. As evidence accumulates, these multimodal strategies are likely to become an integral part of personalised treatment plans in clinical practice.

## Surgical options in severe or pathologic myopia

Surgical interventions in myopia are primarily reserved for severe or pathologic cases where refractive correction alone is insufficient and the risk of irreversible vision loss is significant. While surgical procedures are not typically first-line options for controlling axial elongation or halting progression in children, they remain essential in the management of advanced myopic pathology, especially in adults with degenerative changes. The two main categories of surgical management include refractive correction procedures and posterior scleral reinforcement (PSR) aimed at stabilising the globe structurally [71, 72].

Refractive surgeries such as LASIK (laser-assisted in situ keratomileusis), SMILE (small-incision lenticule extraction), and photorefractive keratectomy (PRK) are commonly used to correct the refractive error associated with myopia in adults [73]. These procedures reshape the cornea to shift the focal point back onto the retina, providing independence from glasses or contact lenses. Although effective in correcting refractive error, these techniques do not address the underlying cause of myopia—namely, axial elongation—and therefore do not prevent disease progression or associated complications such as myopic maculopathy, choroidal neovascularization, or retinal detachment. Their role in children is also limited due to ongoing ocular growth and the higher risks associated with surgical intervention in younger patients.

In cases of very high or pathological myopia, posterior scleral reinforcement (PSR) has emerged as a surgical approach specifically aimed at limiting axial elongation. PSR involves placing a biocompatible graft—such as donor sclera or synthetic material—on the posterior pole of the eye to provide mechanical support and reduce further expansion of the globe. The procedure is designed to stabilise the scleral wall and slow the progression of myopia, particularly in individuals with progressive posterior staphyloma or chorioretinal atrophy. Studies have demonstrated that PSR can reduce the rate of axial elongation and delay the progression of degenerative retinal changes in selected patients, especially when performed in the early stages of pathologic remodelling [72].

Advancements in PSR materials and techniques, such as the use of genipin-crosslinked donor sclera and customised grafts, have improved surgical outcomes and reduced complication rates. However, the procedure remains technically demanding and is associated with potential risks, including globe perforation, haemorrhage, and postoperative fibrosis. As a result, PSR is typically reserved for highly myopic patients with documented progressive pathology, rather than for routine myopia management [74].

Implantable collamer lenses (ICLs) and refractive lens exchange (RLE) are additional surgical options used in adults with high myopia who are not suitable candidates for corneal refractive surgery. ICLs provide effective vision correction by implanting a phakic lens into the posterior chamber of the eye without altering the cornea. RLE involves replacing the natural crystalline lens with an intraocular lens (IOL), a procedure analogous to cataract surgery. Both approaches are refractive in nature and do not halt progression, but they offer high visual quality and may be appropriate in patients with thin corneas or extreme myopia [75]. In conclusion, surgical interventions have a defined but limited role in the comprehensive management of myopia. While they provide effective refractive correction or structural stabilisation in advanced cases, they are not substitutes for early preventive strategies. Careful patient selection and long-term monitoring are essential to optimise outcomes and minimise surgical risk.

## Comparative efficacy and practical considerations

As the landscape of myopia management expands, it becomes increasingly important for clinicians and researchers to understand the relative efficacy, safety, and practicality of available interventions. While several therapeutic options—optical, pharmacological, behavioural, and emerging—have demonstrated measurable success in slowing myopia progression, each carries unique advantages and limitations that must be considered within the context of individual patient needs, age, and risk profile.

Low-dose atropine remains one of the most effective pharmacologic interventions. Clinical trials such as ATOM and LAMP have shown that 0.01–0.05% atropine can reduce myopia progression by 30–70%, with the greatest efficacy observed at the 0.05% concentration. Importantly, the treatment is generally well tolerated, especially at lower concentrations, with minimal impact on pupil size or accommodation. However, variability in response across ethnic groups and the potential for rebound after abrupt cessation necessitate close monitoring and gradual tapering [76].

Orthokeratology (Ortho-K) lenses offer 30–60% reduction in axial elongation, with strong evidence from randomised controlled trials supporting their use in children with moderate myopia. The main advantages include effective control during sleep and daytime freedom from corrective lenses. However, Ortho-K requires rigorous hygiene practices, frequent follow-up, and may not be suitable for younger children due to the small but notable risk of microbial keratitis [77].

Multifocal soft contact lenses (MFCLs), including high-add and EDOF designs, provide similar control to Ortho-K, particularly in cooperative older children and adolescents. Trials such as the BLINK study reported up to 43% reduction in progression over three years. Compared to spectacles, MFCLs offer better peripheral defocus control but may be less practical for children who struggle with lens handling or have contraindications to contact lens wear [78].

Spectacle-based interventions, such as DIMS and HALT-based lenses (e.g., Stellest™), offer safer, easier-to-administer alternatives. These lenses are particularly well suited for younger children or those unable to tolerate contact lenses. Though slightly less effective than MFCLs or Ortho-K in most head-to-head comparisons, spectacle lenses still achieve reductions of up to 50% in myopia progression and have excellent adherence profiles.

Repeated low-level red-light (RLRL) therapy is an emerging approach that may rival or exceed the efficacy of existing interventions. Clinical trials have shown RLRL to reduce axial elongation comparably or more than established methods, with minimal adverse effects. However, device availability, treatment logistics, and long-term outcomes remain under investigation.

Behavioural interventions, especially increasing outdoor time, are strongly recommended for delaying myopia onset, though their effect on progression in already myopic children is less conclusive. Nonetheless, they are low-risk, low-cost, and easily incorporated into public health policies and school programs. In practice, many clinicians are moving toward personalised, multimodal approaches, combining therapies such as low-dose atropine with Ortho-K or MFCLs to enhance efficacy. These combinations have shown additive benefits in several studies, particularly for children with high baseline risk or rapid progression. Ultimately, treatment decisions should be individualised, balancing efficacy, safety, child age, lifestyle, and family preferences. Regular monitoring, clear communication with parents, and adherence to evidence-based guidelines are essential to achieving optimal outcomes in contemporary myopia management. Key randomised controlled trials investigating myopia control therapies are summarised in Table 2, highlighting variations in efficacy, population, and study design across major interventions.

**Table 2:** Comparative efficacy across major myopia control trials.

Trial Name	Intervention studied	Study Population	Duration	Axial Elongation reduction	Notable Findings
ATOM 1	Atropine 1%	Singaporean children (6–12 years)	2 years	~77%	High efficacy but significant side effects with 1%
ATOM 2	Atropine 0.5%, 0.1%, 0.01%	Singaporean children (6–12 years)	2 years	0.01%: ~50%, 0.1%: ~60%, 0.5%: ~65%	0.01% had fewest side effects but lower rebound control
LAMP (Phase 1 & 2)	Atropine 0.01%, 0.025%, 0.05%	Hong Kong children (4–12 years)	2 years	0.05%: ~67%, 0.025%: ~50%, 0.01%: ~34%	0.05% most effective with tolerable side effects
LAMP (5-Year)	Atropine 0.05%	Hong Kong children (8–13 years)	5 years	~55% (vs control)	Sustained efficacy with low rebound at 0.05%
BLINK	Multifocal Soft Contact Lenses (MFCLs)	US children (7–11 years)	3 years	~43%	High-add lenses superior to single-vision for progression control
ROMIO	Orthokeratology (Ortho-K)	Chinese children (6–10 years)	2 years	30–60%	Effective non-pharma method, requires compliance
RLRL Trials (JAMA 2022 & 2023)	Repeated Low-Level Red-Light Therapy	Chinese children (7–13 years)	6–12 months	60–75%	Emerging high-efficacy non-invasive option

## Challenges, gaps, and future directions

Despite the significant progress made in understanding and managing myopia, several challenges persist that hinder the widespread implementation of effective control strategies and limit long-term success. These challenges span scientific, clinical, logistical, and public health domains, underscoring the need for continued research, policy development, and innovation. One of the foremost challenges is the lack of consensus on optimal treatment algorithms. While numerous studies have demonstrated the efficacy of specific interventions such as low-dose atropine, orthokeratology, and multifocal contact lenses, there is still no universally accepted protocol regarding when to initiate treatment, how to combine modalities, and when to discontinue therapy. Most clinical decisions are guided by practitioner experience rather than standardised, evidence-based algorithms. Additionally, interindividual variability in response—driven by age, ethnicity, environmental factors, and genetic predisposition—makes it difficult to predict outcomes and tailor treatments effectively.

Another limitation is the short duration of most clinical trials, which typically range from one to three years. Given that myopia often progresses over a decade or more, long-term safety and durability of effect remain uncertain. Questions about the sustainability of treatment benefits, especially after cessation of therapy, are particularly relevant for interventions like low-dose atropine and RLRL therapy, where rebound effects or regression may occur. Furthermore, many studies are geographically concentrated in East Asia, with limited data from Western, African, or multi-ethnic populations, restricting the generalisability of findings. Adherence and compliance also present ongoing barriers, particularly among children. Treatments such as Ortho-K or contact lens wear demand strict hygiene and regular follow-up, which may not be feasible for all families. Even simple interventions like daily atropine eye drops or increased outdoor activity can suffer from inconsistent adherence, especially without strong parental engagement or school-based support.

From a public health perspective, access and affordability of advanced interventions remain critical issues. Technologies like RLRL devices or custom-designed myopia control lenses may not be readily available in low- and middle-income settings. The cost of long-term treatments can also be prohibitive for many families, particularly in regions where these interventions are not covered by insurance or public health programs. This has the potential to widen disparities in vision outcomes globally. Moreover, there is a paucity of data on myopia prevention strategies in early childhood, before refractive error becomes measurable. Interventions during the pre-myopic stage—such as outdoor programs in preschools or early parental education—could prove valuable but remain underexplored. Similarly, little is known about how digital media exposure, sleep patterns, and urban environmental factors contribute to early-onset myopia, despite their likely influence.

Looking forward, future research should focus on individualised risk prediction models, integrating axial length data, genetic markers, and behavioural risk profiles to guide early intervention. There is also an urgent need for interdisciplinary collaboration between ophthalmologists, optometrists, educators, and policymakers to implement scalable preventive strategies, particularly in school settings. In conclusion, while significant strides have been made in slowing myopia progression, addressing these multifaceted challenges is essential for transitioning from treatment of individuals to effective population-level myopia control. A proactive, global approach that prioritises equity, education, and early intervention will be critical to mitigate the long-term consequences of this growing public health concern.

## Conclusion

Myopia has become a global public health concern, with rising prevalence and increasing risk of vision-threatening complications in high and pathologic cases. Over the last two decades, multiple interventions have been developed to slow progression, including optical strategies (e.g., multifocal lenses, orthokeratology), pharmacologic treatments (notably low-dose atropine), behavioural modifications, and emerging options like repeated low-level red-light therapy. While these interventions show promising results, there is no single solution applicable to all patients. Individualised, evidence-based approaches—often combining multiple strategies—offer the best chance of effective control. However, challenges remain in adherence, access, long-term data, and population-wide implementation. Future success will depend on early intervention, public health engagement, and multidisciplinary collaboration. A proactive, personalised approach is key to reducing the global burden of myopia and preserving visual function across the lifespan.

### Literature search strategy

This narrative review is based on a comprehensive, non-systematic search of English-language literature published between 2000 and May 2025. Sources included PubMed, Google Scholar, ScienceDirect, and the Cochrane Library. Keywords such as “myopia control,” “axial elongation,” “orthokeratology,” “low-dose atropine,” “red-light therapy,” and “outdoor activity” were used. Preference was given to randomised controlled trials, meta-analyses, and high-impact reviews relevant to paediatric myopia management. Articles were selected based on relevance, originality, and clinical significance. The review reflects the authors’ synthesis of the most current and influential evidence available.

## Summary

### What is known about this topic

- Myopia is rising globally, particularly among school-aged children, and is associated with long-term vision-threatening complications in high and pathologic forms.
- Interventions such as low-dose atropine, orthokeratology, and multifocal soft contact lenses have demonstrated efficacy in slowing axial elongation and refractive progression.
- Outdoor activity is widely recommended as a preventive strategy, though its effect on slowing progression after onset remains limited.
- Repeated low-level red-light (RLRL) therapy has emerged as a non invasive intervention with encouraging short-term results.

### What this study adds

- Offers an up-to-date narrative review synthesizing current and emerging, myopia control strategies, including novel interventions such as RLRL and combination therapies.
- Compares intervention efficacy, safety, and practical application based on recent clinical trials and meta-analyses.
- Underscores the benefits of combining pharmacological and optical treatments in managing progressive myopia, particularly in highrisk pediatric populations.
- Identifies critical barriers to long-term success—such as variable adherence, limited access, and lack of standardized treatment pathways—and proposes future directions for individualized, integrated myopia care.

## Data Availability

This is a narrative review based on previously published literature. No new datasets were generated or analysed during the current study. All data supporting the findings are available within the cited references.
